# High Incidence of Epileptiform Potentials During Continuous EEG Monitoring in Critically Ill COVID-19 Patients

**DOI:** 10.3389/fmed.2021.613951

**Published:** 2021-03-26

**Authors:** Barbara Schultz, Terence Krauß, Maren Schmidt, Michael Schultz, Andrea Schneider, Olaf Wiesner, Julius J. Schmidt, Klaus Stahl, Sascha David, Marius M. Hoeper, Markus Busch

**Affiliations:** ^1^Department of Anesthesiology and Intensive Care Medicine, Hannover Medical School, Hannover, Germany; ^2^Medical University of Vienna, Vienna, Austria; ^3^Department of Gastroenterology, Hepatology and Endocrinology, Hannover Medical School, Hannover, Germany; ^4^Department of Respiratory Medicine and German Centre of Lung Research (DZL), Hannover Medical School, Hannover, Germany; ^5^Department of Nephrology and Hypertension, Hannover Medical School, Hannover, Germany; ^6^Institute of Intensive Care Medicine, University Hospital Zurich, Zurich, Switzerland

**Keywords:** COVID-19, electroencephalography, critical care, epileptiform potentials, inhalation anesthetics, sedation

## Abstract

**Objective:** To analyze continuous 1- or 2-channel electroencephalograms (EEGs) of mechanically ventilated patients with coronavirus disease 2019 (COVID-19) with regard to occurrence of epileptiform potentials.

**Design:** Single-center retrospective analysis.

**Setting:** Intensive care unit of Hannover Medical School, Hannover, Germany.

**Patients:** Critically ill COVID-19 patients who underwent continuous routine EEG monitoring (EEG monitor: Narcotrend-Compact M) during sedation.

**Measurements and Main Results:** Data from 15 COVID-19 patients (11 men, four women; age: 19–75 years) were evaluated. Epileptiform potentials occurred in 10 of 15 patients (66.7%).

**Conclusions:** The results of the evaluation regarding the occurrence of epileptiform potentials show that there is an unusually high percentage of cerebral involvement in patients with severe COVID-19. EEG monitoring can be used in COVID-19 patients to detect epileptiform potentials.

## Introduction

While the pulmonary manifestation of severe acute respiratory syndrome coronavirus-2 (SARS-CoV-2) is now well-described, there is a lot less clarity regarding other organic manifestations (1). Extrapulmonary organic complications, including cardiac, thromboembolic and gastrointestinal manifestations, are increasingly acknowledged as important and potentially severe complications of coronavirus disease 2019 (COVID-19) (2–4). There have also been systematic reports of neurological involvement and symptoms associated with SARS-CoV-2 infection in critically ill patients. A study from Wuhan documented neurological symptoms in 78 (36.4%) of 214 patients hospitalized with COVID-19 (5). A French study found neurological symptoms in 8 (14%) of 58 patients admitted to the intensive care unit, and in 39 (67%) after the sedation had ended (6).

At present, only little data is available regarding EEG findings of patients with COVID-19 (7). For intensive care patients requiring invasive mechanical ventilation with a severe course of COVID-19 disease, examination methods such as computed tomography (CT), cranial magnetic resonance imaging (cMRI) or routine electroencephalogram (EEG) are only available to a limited extent due to the contagiousness of the virus and the fact that a prone position is often necessary. Furthermore, routine electroencephalogram (EEG) is also only available to a limited extent within routine critical care due to the fact that multi-channel EEG-devices can be difficult to decontaminate (8). However, 2-channel EEG monitoring can be carried out as a bedside procedure and may be used not only to assess the depth of sedation, but also to diagnose seizures early that might otherwise go clinically unnoticed, and to then initiate prompt treatment (9).

In this paper, continuous 1- or 2-channel EEGs from SARS-CoV-2-infected intensive care patients requiring ventilation were evaluated retrospectively. The EEGs were analyzed with regard to the depth of sedation at the beginning of the recording and with regard to the occurrence of epileptiform potentials throughout the EEG.

## Materials and Methods

Between March and July 2020, EEG monitoring was performed on 15 consecutive critically ill patients requiring mechanical ventilation and sedation due to COVID-19-associated acute respiratory distress syndrome (ARDS). Polymerase chain reaction (PCR) testing was performed repeatedly in all patients. Fourteen patients each had at least two PCR tests confirming SARS-CoV-2 infection. Seven of these patients were also tested for antibodies against SARS-CoV-2 with positive results in six patients. In one patient, the diagnosis of COVID-19 was made on the basis of repeated positive antibody tests and on the basis of the clinical picture.

Inhaled anesthetics were given in addition to intravenous hypnotics to achieve deep levels of sedation, if necessary. The choice of substance was at the discretion of the attending doctors. Inhaled anesthetics were administered using AnaConDa® (Sedana medical, Two Mile House, Ireland) as standard.

Multi-channel EEG machines were not available for the COVID-19 patients because of the difficulty of device decontamination. Instead, 2-channel EEG devices were installed at the bedside. Continuous 1- or 2-channel EEG monitoring is routinely performed in other intensive care units in the hospital, e.g., in a cardiac intensive care unit and a pediatric intensive care unit. The staff of the ICU with COVID-19 patients was extensively trained in the use of the EEG monitors and in the interpretation of the EEG signals. The EEGs were used to monitor the depth of sedation and to detect early signs of cerebral involvement.

**Figure 1:**
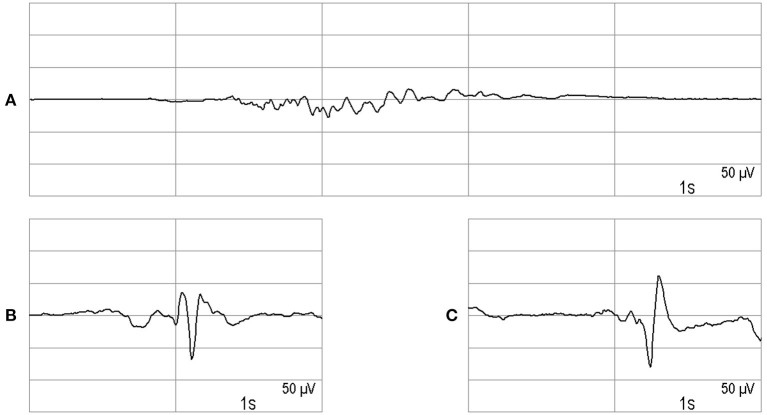
**(A)** Burst suppression. **(B,C)** Epileptiform potentials recorded in two patients.

EEG monitoring was usually carried out as a 2-channel recording, by way of exception also as a 1-channel recording. The EEG electrodes were placed on the forehead or the temple (EEG monitor: Narcotrend®-Compact M, MT MonitorTechnik GmbH & Co. KG, Bad Bramstedt, Germany). The monitor automatically evaluates the EEG with regard to the depth of sedation using a scale from A (awake) to F_1_ (very deep hypnosis) (10). In the stages A to E_1_, the EEG is continuous, i.e., without suppression periods, and shows a progressive slowing from stage B to E_1_. The stages D_0_ to E_1_ are characterized by an increasing amount of delta waves (0.5–3.5 Hz). Stage E_2_ is a transition stage to the burst suppression pattern, which is characterized by intermittent very flat periods. In the stages F_0_-F_1_, there are suppression periods of increasing length up to a continuous suppression. Figure 1A shows an example of stage F_0_.

EEG monitoring was usually started when the patients were sedated, intubated and ventilated. It was generally stopped when the patients became clinically more awake toward the end of the sedation phase. In one case, the EEG was reconnected because the patient suffered a clinical seizure with an ensuing altered state of consciousness. The duration of the monitoring was between 2.1 and 29.9 days and all recorded original EEG data were available for analysis. They were visually evaluated in full with regard to epileptiform potentials. The person assessing the EEGs was not blinded to the patients' COVID-19 status.

In our analysis, we wanted to evaluate if any epileptiform discharges occurred. We use the term epileptiform potentials for wave forms of epileptiform interictal activity (11), including those occurring as part of periodic or rhythmic patterns (12), and for ictal activity consisting of epileptiform discharges or rhythmic discharges (11, 13). Figures 1B,C show examples of epileptiform potentials.

To evaluate a potential association between the occurrence of epileptiform potentials and inflammatory biomarkers, C-reactive protein (CRP) and interleukin-6 (IL-6) concentrations were analyzed in COVID-19 patients with and without epileptiform potentials.

All personal patient data were anonymized before further analysis. Data was collected using electronic medical records including the patient data monitoring system (PDMS) m.life (Version 10.5.0.71, medisite GmbH, Berlin, Germany). The statistical software SAS (version 9.3, SAS Institute, Cary, USA) was used for data analysis. The Wilcoxon test was used to compare mean values.

The study was performed in accordance with the ethical standards laid down in the 1964 Declaration of Helsinki and its later amendments. The ethics committee of Hannover Medical School had approved the retrospective evaluation of EEGs performed in intensive care patients in routine clinical practice.

## Results

Data from 15 patients with COVID-19 was included in the evaluation, comprising 11 men and four women aged 19–75 years with body weights between 70 and 130 kg (body mass index, BMI, range 24–47).

At the start of the EEG recording, 12 patients received an inhalation anesthetic (11 isoflurane, 1 sevoflurane) at minimum alveolar concentrations (MAC) between 0.4 and 1.25. In addition, propofol was administered to 11 of the 12 patients (between 0.31 and 2.86 mg/kg/h). All 12 patients received sufentanil (between 0.05 and 0.21 μg/kg/h); one patient was also given esketamine (2 mg/kg/h). Three patients were sedated without an inhalation anesthetic. These patients received propofol and sufentanil and additionally esketamine (one patient) or dexmedetomidine (one patient). Ten of the 15 patients had an EEG stage in the E_2_-F_1_ range at the start of the EEG recording; in these 10 patients, an inhalation anesthetic was part of the analgosedation.

In this cohort of 15 patients, 10 patients demonstrated epileptiform potentials during the course of disease (66.7%). In one of the 10 patients from the group with and in one patient without epileptiform potentials, phases with rhythmic delta waves around 1 Hz occurred.

From the group of patients with epileptiform potentials, one patient displayed a brief episode of myoclonus of the extremities while sedated, and two patients had a generalized tonic-clonic seizure before or during the observation period. In the other patients with epileptiform potentials, no clinical signs suggestive of seizures were noted. A cMRI was performed in two of the patients with epileptiform potentials. Cerebral microbleeds were seen in both cases.

When the epileptiform potentials occurred, the following EEG sedation stages were present in the COVID-19 patients: D_2_ (two patients), E_0_ (one patient), E_2_ (five patients), F_0_ (one patient). In one patient, a sedation stage was not determined because of ongoing epileptiform potentials.

With regard to the levels of CRP and IL-6, there was no statistically significant difference between the two groups of COVID-19 patients with and without epileptiform potentials when considering the values at the start of the EEG measurements. In the patients with or without epileptiform potentials, the CRP values were 138.50 ± 64.24 and 114.20 ± 40.55 mg/L, respectively (mean ± standard deviation, *p* = 0.43). The IL-6 values were 1,023.60 ± 2,434.84 and 596.80 ± 784.36 ng/L, respectively (mean ± standard deviation, *p* = 0.18).

## Discussion

EEG monitoring provided clinically relevant information for the examined COVID-19 patients both with regard to the presence of epileptiform potentials and with regard to the depth of sedation. EEG is sometimes the only way to detect neurological involvement in sedated patients with COVID-19.

Individual authors have reported on EEG abnormalities in COVID-19 patients. Galanopoulou et al. (14) report an investigation of a group of 22 COVID-19 patients, 14 of whom were intubated at the time of the EEG. Sporadic epileptiform discharges were found in 40.9% of the patients (9/22) (14). Somani et al. (15) report on two COVID-19 patients who had experienced a status epilepticus during admission or in the course of intensive therapy, which, as was evident from the EEG, emanated from fronto-central regions in one patient and from fronto-central-parietal regions in the other patient (15). Conspicuous rhythmic EEG activity was described by Vespignani et al. for five out of 22 patients with severe SARS-CoV-2 infections; four of the five patients were intubated and poorly responsive or unresponsive (16).

Rhythmic and periodic EEG patterns can occur in patients with acute brain damage, e.g., traumatic brain injury, cerebral hemorrhage, meningitis or encephalitis, but also sepsis, toxic-metabolic encephalopathy and hypoxic-ischemic encephalopathy (17). Different neuropathological changes in the brain, resembling both vascular and demyelinating etiologies, were found in a patient who died as a result of SARS-CoV-2 infection (18), and other authors also report on vascular cerebral processes in COVID-19 patients (19).

Galanopoulou et al. observed that frontal spikes were the most common epileptiform discharge pattern in COVID-19 patients (14). According to the authors, this pattern suggests a frontal epileptogenic focus or a frontal dysfunction.

In our cohort, 10 of 15 patients (66.7%) had epileptiform potentials in the EEG. This percentage was higher than the proportion of EEGs with epileptiform potentials in the study by Galanopoulou et al. (14) (40.9%). Taking into account the patient with rhythmic delta waves and without epileptiform potentials, 11 of our 15 patients (73.3%) showed abnormalities in the EEG. The percentage (66.7%) of patients with epileptiform potentials is higher than we would have expected based on our own experience with continuous EEG monitoring in ventilated patients without COVID-19 in a medical/anesthesiological ICU.

Serum CRP and IL-6 were not associated with the occurrence of epileptiform potentials in the COVID-19 group. The high incidence can best be explained by pathological changes in the brain or brain function. No history of symptomatic epilepsy was documented in any of our patients.

The frequency with which epileptiform potentials occurred in this study underlines the importance of cerebral monitoring in COVID-19 patients. If convulsive or non-convulsive seizures or a status epilepticus occur, early detection and therapy is necessary to avoid cerebral sequelae (20). Flamand et al. emphasize that it is important to pay more attention to the EEG patterns that occur in patients during the COVID-19 pandemic (21).

Patients with COVID-19 disease have an increased risk of delirium (22). A very deep sedation is a risk factor for delirium after sedation and ventilation (22, 23). On the other hand, seizures can also cause protracted delirium. In the area of anesthesia, the European Society of Anaesthesiology, in a guideline on postoperative delirium, describes intraoperative neuromonitoring as important in order to avoid unnecessarily deep anesthesia, which often reaches burst suppression in older patients (24). In the field of intensive care, it has been shown that the time in burst suppression is an independent predictor of the occurrence and duration of delirium after coma or sedation (23). Especially when combining sedatives, such as inhalation anesthetics and intravenous hypnotics, the sedation depth is difficult to assess based on clinical parameters. Using EEG, the sedation depth can be assessed and adapted to the individual needs.

## Strengths and Limitations

Inhalation anesthetics, i.e., isoflurane or sevoflurane, were administered to most of the patients. In literature, there are very few reports of possible epileptogenic effects of isoflurane in humans (25, 26). In epileptic adult patients, increasing interictal spike frequency in the electrocorticogram was reported to occur with increasing isoflurane concentrations (26), but other authors reported stable or decreasing frequencies during isoflurane administration (27, 28). None of the COVID-19 patients in our study had a history of epilepsy. There have been several reports on the use of isoflurane for the treatment of status epilepticus (29). Contrary to isoflurane, sevoflurane has been frequently reported to induce epileptiform activity in both children and adults (30, 31); caution is required regarding its use in epileptic patients (32). One of the COVID-19 patients in our study received sevoflurane, but the administration period had ended before epileptiform potentials occurred in the EEG of this patient. Four of the 15 patients in our study received esketamine for some time while the EEG was being recorded. Epileptiform potentials were recorded in one patient during ketamine administration; in the other three patients, either no epileptiform potentials occurred, or there was no temporal relationship between esketamine administration and occurrence of epileptiform potentials. The small number of patients does not allow any statement to be made regarding a relationship between esketamine administration and the occurrence of epileptiform potentials.

A strength of our investigation is the use of continuous EEG monitoring. Compared to intermittent EEG examinations, this increases the likelihood of detecting EEG changes (33). The use of a reduced number of channels is advantageous for practical reasons (patient positioning, efforts required to maintain good EEG signal quality), but could lead to a reduced detection of EEG changes (34). The relatively small number of cases in the group of COVID-19 patients can also be seen as a limitation.

## Conclusions

These observations suggest a high incidence of epileptiform potentials in patients with severe COVID-19 on invasive mechanical ventilation. Continuous EEG may be a useful non-invasive tool to monitor these patients. Further studies are required to determine the clinical implications of these observations and to study interventional strategies.

## Data Availability Statement

The raw data supporting the conclusions of this article will be made available by the authors, without undue reservation.

## Ethics Statement

The studies involving human participants were reviewed and approved by Ethics Committee of Hannover Medical School. Written informed consent for participation was not required for this study in accordance with the national legislation and the institutional requirements.

## Author Contributions

BS: study design, EEG analysis, and statistical analysis. TK, MSchm, AS, OW, JS, KS, SD, MH, and MB: clinical data acquisition. BS, MSchu, and MB: literature analysis and drafting the manuscript. BS, TK, MSchm, MSchu, AS, OW, JS, KS, SD, MH, and MB: revising the manuscript. All authors contributed to the article and approved the submitted version.

## Conflict of Interest

The authors declare that the research was conducted in the absence of any commercial or financial relationships that could be construed as a potential conflict of interest.
